# The Journal

**Published:** 1851-04

**Authors:** 


*The Journal.*—Dr. Harris, the present editor, has associated with him, in the management and editorship of the Journal, Alfred A. Blandy, M. D., who will hereafter assist in discharging all duties connected with the publication.

